# Dietary changes in the NutriNet Brasil cohort during the covid-19 pandemic

**DOI:** 10.11606/s1518-8787.2020054002950

**Published:** 2020-08-28

**Authors:** Eurídice Martínez Steele, Fernanda Rauber, Caroline dos Santos Costa, Maria Alvim Leite, Kamila Tiemann Gabe, Maria Laura da Costa Louzada, Renata Bertazzi Levy, Carlos Augusto Monteiro

**Affiliations:** I Universidade de São Paulo Núcleo de Pesquisas Epidemiológicas em Nutrição e Saúde São PauloSP Brasil Universidade de São Paulo . Núcleo de Pesquisas Epidemiológicas em Nutrição e Saúde da USP (NUPENS). São Paulo , SP , Brasil; II Universidade de São Paulo Faculdade de Medicina Departamento de Medicina Preventiva São PauloSP Brasil Universidade de São Paulo . Faculdade de Medicina . Departamento de Medicina Preventiva . São Paulo , SP , Brasil; III Universidade de São Paulo Faculdade de Saúde Pública Departamento de Nutrição São PauloSP Brasil Universidade de São Paulo . Faculdade de Saúde Pública . Departamento de Nutrição . São Paulo , SP , Brasil

**Keywords:** Coronavirus infections, Food Consumption, Whole Foods, Industrialized Foods

## Abstract

**OBJECTIVE:**

To describe the dietary characteristics of participants in the NutriNet Brasil cohort immediately before and during the covid-19 pandemic.

**METHODS:**

Our data stem from an adult cohort created to prospectively investigate the relationship between diet and morbidity and mortality from chronic non-communicable diseases in Brazil. For this study, we selected the first participants (n = 10,116) who answered twice to a simplified questionnaire on their diet the day before, the first time when entering the study, between January 26 and February 15, 2020, and the second between May 10 and 19, 2020. The questionnaire inquiries about the consumption of healthy (vegetables, fruits and legumes) and unhealthy (ultra-processed foods) eating markers. Comparisons of indicators based on the consumption of these markers before and during the pandemic are presented for the study population and according to gender, age group, macro-region of residence and schooling. Chi-square tests and t-tests were used to compare proportions and means, respectively, adopting p < 0.05 to identify significant differences.

**RESULTS:**

For all participants, we found a modest but statistically significant increase in the consumption of healthy eating markers and stability in the consumption of unhealthy food markers. This favorable pattern of dietary changes during the pandemic occurred in most sociodemographic strata. We observed a less favorable changing pattern, with a tendency to increasing consumption of healthy and unhealthy food markers, in the Northeast and North macro-regions and among people with less schooling, suggesting social inequalities in the response to the pandemic.

**CONCLUSIONS:**

If confirmed, the trend of increased consumption of ultra-processed foods in underdeveloped regions and by people with less schooling is concerning, as eating these foods increases the risk of obesity, hypertension and diabetes, whose presence increases the severity and lethality of covid-19.

## INTRODUCTION

The covid-19 pandemic, an infection caused by the new Sars-CoV-2 coronavirus, officially arrived in Brazil on February 26, 2020. In mid-March, due to the geometric increase in the number of cases and the lack of vaccine or effective treatments for the disease, health authorities in Brazilian states and municipalities started measures already implemented in other countries, seeking to substantially reduce interpersonal contact. These actions included stopping all non-essential economic activities, closing schools, and express recommendations for people to stay home as long as possible. Estimates of May 10, based on data from mobile phone operators in Brazil, indicated that about half of people followed the recommendations to stay home ^1^ .

It is reasonable to expect that many behaviors will change with the covid-19 pandemic, one of which is diet. Several factors associated with the pandemic could influence food quality, both positively and negatively. The greater number of people staying at home, together with the closure of bars, restaurants and other businesses that serve food or meals for consumption on site, predicts a large increase in the proportion of meals cooked at home, which would be positive if we consider that homemade food tends to be healthier than food eaten away from home ^2^ . Another factor that could contribute to improving diet would be an eventual greater concern in consuming healthier foods as a way of increasing the immune defenses against a disease for which no vaccine or effective treatment still exists. Factors that could lead to less healthy eating behaviors would include greater difficulty in obtaining fresh foods, whose acquisition requires more frequent departures from home, and the eventual reduction in family income due to job loss or impossibility to perform certain occupations, limiting the purchase of higher-priced foods, such as fruits and vegetables. It should be highlighted that the influence of all these factors on eating behavior can be modulated by populations’ and individuals’ cultural, educational and economic characteristics.

Many reasons justify studying the impact of the covid-19 pandemic on the population’s eating behavior, including the unpredictability of the duration of the pandemic, and of the measures implemented for its control, and the influence that diet has on chronic non-communicable diseases, which increase the severity and lethality of covid-19 ^3^ .

In this article we describe dietary characteristics of participants in the NutriNet Brasil cohort immediately before the pandemic reached the country and during the pandemic.

## METHODS

All data from this study stem from the NutriNet Brasil cohort, created to prospectively investigate the relationship between dietary patterns and morbidity and mortality from chronic non-communicable diseases in Brazil ^4^ . Participation in the cohort is voluntary, and participant recruitment – people residing in any state of the Federation of at least 18 years of age – began on January 26, 2020 through invitations disclosed by regional and national mass media and by social media influencers with tens or hundreds of thousands of followers throughout Brazil. The cohort already has more than 75,000 respondents from all states of the Federation. Participants register on the study’s digital platform ^4^ and, every three months, on the same website, answer questionnaires addressing their health status and diet, in addition to other conditions that can influence health.

For this study, we selected the first participants of the NutriNet Brasil cohort who answered twice to a simplified questionnaire on their diet the day before, the first time when entering the study, between January 26 and February 15, 2020 (i.e., before the covid-19 pandemic reached Brazil), and the second between May 10 and 19, 2020 (i.e., during the pandemic). We extracted the information provided by the participants (n = 10,116) from the cohort database on May 19, 2020.

The simplified food questionnaire is an adaptation of an instrument developed by the authors for the Ministry of Health Surveillance of Risk and Protective Factors for Chronic Diseases by Telephone Survey (Vigitel) system ^5^ , which has also been used in two national surveys from the Brazilian Institute of Geography and Statistics (IBGE), the *Pesquisa Nacional de Saúde* (PNS 2019 – National Health Survey) ^6^ and the *Pesquisa Nacional de Saúde do Escolar* (PeNSE 2019 – National Student Health Survey) ^7^ . The questionnaire asks whether, on the day before, participants consumed (yes or no) foods selected as markers of healthy eating or unhealthy eating. Only from the sixth month of follow-up onwards will participants of the NutriNet Brasil cohort answer complete 24-hour dietary recalls.

The list of healthy eating markers in the simplified questionnaire of the NutriNet Brasil cohort includes 29 items: 18 types of vegetables (lettuce, chard, watercress, arugula, collard, cabbage, broccoli, spinach, any other greens, tomato, cucumber, carrot, beetroot, pumpkin, zucchini, eggplant, okra and any other vegetable), 10 types of fruit (banana, orange or tangerine, mango, papaya, pineapple, watermelon or melon, apple or pear, grape, açaí, any other fruit), in addition to beans or other legumes (lentil and chickpea).

The list of unhealthy food markers includes 23 groups of ultra-processed foods: soda, bottled or canned juice, fruit drink prepared from powdered mix, chocolate milk drink, tea-based drink, flavored yogurt, sausage or hamburger or nuggets, ham or salami or bologna, buns, rolls or any type of packaged breads, margarine, frozen french fries, mayonnaise or ketchup or mustard, ready-to-eat salad dressing, instant noodles or instant powder soup, frozen or chain pizza, frozen lasagna or other frozen ready-to-eat meals, potato chips or packaged salty snacks or crackers, cookies with or without filling, branded (not homemade or artisanal) cakes, muffins or sweet pies, cereal bar, ice cream or popsicles, chocolate bar or chocolate candy, and sugared breakfast cereals.

Based on the answers given by the cohort participants on their food intake the day before, we calculated four healthy eating indicators – percentage of people who consumed vegetables (of any type); fruit (of any kind); beans or other legumes; and all three previous items – and three unhealthy eating indicators – percentage of people who consumed at least one group of ultra-processed foods; five or more groups of ultra-processed foods; and average number of ultra-processed food groups consumed.

Comparisons between estimates of indicators before and during the pandemic are presented for the whole population studied and for strata defined by gender (male and female), age group (18-39, 40-59 and > 60 years), macro-region of residence (North, Northeast, Midwest, Southeast and South) and schooling (< 11, 11 and > 12 full years of study). Statistical tests based on chi-square distribution were used for comparing proportions, and t-tests for comparing means, adopting p < 0.05 values to identify significant differences.

## RESULTS

Table 1 presents sociodemographic characteristics of the 10,116 participants in the NutriNet Brasil cohort whose diet before and during the covid-19 pandemic we describe in this study. We observed a predominance of females (78.0%), young adults (51.1%), residing in the Southeast macro-region (61.2%) and people with 12 or more years of schooling (85.1%). The absolute number of participants is particularly low, falling below 1,000 among older adults (n = 908), residents of the Midwest (732) and North (290) macro-regions and people with less than 11 years of schooling (227).

**Table 1:** Distribution according to sociodemographic variables of participants of the NutriNet Brasil Cohort with complete food intake assessments before a and during b the covid-19 pandemic in Brazil.

Variables	N	%
Gender		
Male	2,221	22.0
Female	7,895	78.0
Age group (years)		
18–39	5,174	51.1
40–59	4,034	39.9
> 60	908	9.0
Macro-region of residence		
North	290	2.9
Northeast	1,122	11.1
Midwest	732	7.2
Southeast	6,188	61.2
South	1,784	17.6
Schooling level (years)		
< 11	227	2.2
11	1,283	12.7
> 12	8,606	85.1
**Total**	**10,116**	**100**

^a^ Between 26 January and 15 February 2020.

^b^ Between 10 and 19 May 2020.

### Changes in Healthy Eating Indicators

Table 2 shows estimates of healthy eating indicators in the period immediately preceding the arrival of the covid-19 pandemic in Brazil and during the pandemic. The estimates are provided for the overall group of participants studied and according to sociodemographic strata.

**Table 2:** Healthy eating indicators before a and during b the covid-19 pandemic in Brazil according to sociodemographic variables among participants in the NutriNet Brasil cohort (n = 10,116).

Variables	Consumption the day before of:							
	Vegetables (%)		Fruit (%)		Beans or other legumes (%)		Vegetables + fruit + legumes (%)	
	Before	During	Before	During	Before	During	Before	During
Gender								
Male	85.5	88.7 ^c^	76.7	81.3 ^c^	60.6	63.0	43.9	50.5 ^c^
Female	87.8	89.2 ^c^	78.8	81.9 ^c^	51.4	53.1 ^c^	39.1	43.0 ^c^
Age group (years)								
18–39	85.2	86.3	72.1	75.7 ^c^	56.1	56.3	38.8	42.3 ^c^
40–59	88.9	91.4 ^c^	83.2	86.9 ^c^	50.9	54.3 ^c^	41.1	46.5 ^c^
> 60	92.5	94.5	92.2	93.5	49.8	54.6 ^c^	44.4	49.9 ^c^
Macro-region of residence								
North	84.5	86.9	71.4	76.9	53.4	56.2	36.5	42.8
Northeast	81.2	85.6 ^c^	76.6	80.4 ^c^	63.1	63.6	43.5	47.6
Midwest	89.7	88.9	81.0	79.8	59.4	58.3	46.7	45.8
Southeast	87.8	89.5 ^c^	77.8	82.2 ^c^	53.6	56.0 ^c^	40.2	45.9 ^c^
South	89.2	90.2	81.2	82.8	44.6	46.2	36.3	38.1
Schooling level (years)								
< 11	81.5	85.5	75.8	81.9	64.3	62.9	42.3	49.8
11	83.4	86.9 ^c^	73.0	79.9 ^c^	57.3	61.2 ^c^	38.4	48.2 ^c^
> 12	88.1	89.5 ^c^	79.2	82.0 ^c^	52.6	54.2 ^c^	40.4	43.9 ^c^
Total	87.3	89.1 ^c^	78.3	81.8 ^c^	53.5	55.3 ^c^	40.2	44.6 ^c^

^a^ Between 26 January and 15 February 2020.

^b^ Between 10 and 19 May 2020.

^c^ p < 0.05

For the overall cohort of participants, the four healthy eating indicators evolved favorably. Statistically significant increases, albeit of small magnitude, occur for the frequency of consumption on the day before of vegetables (from 87.3% to 89.1%), of fruits (from 78.3% to 81.8%), of beans or other legumes (53.5% to 55.3%) and the three previous items (from 40.2% to 44.6%).

Most strata showed the same favorable pattern of changes in the four healthy eating indicators, although the variations did not always reach statistical significance. Statistically significant changes in at least one healthy eating indicator were observed among men and women, in young adults (18-39 years), middle age (40-59 years) and older adults ( > 60 years), in the Southeast and Northeast macro-regions and in the categories of intermediate (11 years) and higher ( > 12 years) education.

### Changes in Unhealthy Eating Indicators

Table 3 shows the unhealthy eating indicators before and during the covid-19 pandemic in Brazil.

**Table 3:** Unhealthy eating indicators before a and during b the covid-19 pandemic in Brazil according to sociodemographic variables among participants in the NutriNet Brasil cohort (n = 10,116).

Variables	Consumption the day before of ultra-processed foods					
	Consumption of at least one group (%)		Consumption of five or more groups (%)		Number of groups consumed (average)	
	Before	During	Before	During	Before	During
Gender						
Male	82.3	83.9	14.3	14.0	2.4	2.4
Female	79.3	79.3	10.0	9.4	2.1	2.1
Age group (years)						
18–39	82.8	83.6	12.9	12.6	2.3	2.3
40–59	78.0	77.8	9.3	8.7	2.0	2.0
> 60	72.8	73.2	6.7	5.6	1.7	1.8
Macro-region of residence						
North	77.6	81.7	14.1	14.1	2.2	2.4
Northeast	77.9	79.6	8.8	10.9	2.0	2.2 ^c^
Midwest	77.5	76.2	7.8	7.4	1.9	1.9
Southeast	80.4	80.7	11.5	10.5	2.2	2.2
South	81.3	80.9	11.3	10.2	2.2	2.2
Schooling level (years)						
< 11	86.3	87.2	14.5	15.4	2.5	2.7
11	82.4	83.9	14.1	13.5	2.4	2.4
> 12	79.5	79.6	10.4	9.8	2.1	2.1
Total	80.0	80.3	11.0	10.4	2.1	2.1

^a^ Between 26 January and 15 February 2020.

^b^ Between 10 and 19 May 2020.

^c^ p < 0.05

For the overall cohort of participants, the unhealthy eating indicators hardly changed with the pandemic. Thus, the proportion of participants who consumed at least one group of ultra-processed foods the day before ranged from 80.0% to 80.3%, and five or more groups from 11.0% to 10.4%, while the average number of groups consumed (2.1) remains unchanged.

The pattern of stability in the unhealthy eating indicators was observed among men and women, in all age groups, in the Southeast, South and Midwest macro-regions and in the intermediate and higher schooling categories. Among participants of the Northeast macro-region, the frequency of consumption of at least one group of ultra-processed foods (from 77.9% to 79.6%) and of five or more groups (from 8.8% to 10.9%) increased, as well as the average number of groups consumed (from 2.0 to 2.2), with the latter variation being statistically significant. Although without reaching statistical significance, we observed the same trend of increasing consumption of ultra-processed foods in the North macro-region and in the lower category of schooling, strata in which, for example, the average number of groups of ultra-processed foods consumed increased from 2.2 to 2.4 and from 2.5 to 2.7, respectively.

## DISCUSSION

Based on questionnaires on food consumption the day before answered by just over 10,000 participants in the NutriNet Brasil cohort, immediately before and during the covid-19 pandemic in the country, we identified a modest, but statistically significant increase in the consumption of healthy food markers (vegetables, fruits and legumes) and stability in the consumption of unhealthy food markers (ultra-processed foods). This favorable pattern of dietary changes with the pandemic occurred in most of the cohort strata, defined according to gender, age, macro-region of residence and schooling. In the underdeveloped Northeast macro-region, we observed a less favorable evolution pattern, with significant increases both in the frequency of consumption of vegetables and fruits and in the number of ultra-processed food groups ingested. Although it reached no statistical significance, possibly due to the reduced number of participants, we observed this same pattern of less favorable dietary evolution in the North macro-region, which is also less economically developed, and in the lower schooling category, suggesting social inequalities in the eating behavior response to the pandemic.

It should be noted that, although the invitations to participate in the NutriNet Brasil cohort were disclosed by regional and national mass media and by social media influencers, its first participants present a sociodemographic profile quite different from that expected for the adult Brazilian population. When compared with the expected frequencies according to the 2010 Demographic Census ^8^ , in addition to a smaller proportion of men (22.0% in this study, against 48.2% in the Census) and older adults (9.0% versus 15.3%), the analysis revealed a marked underrepresentation of the Northeast (11.1% versus 26.6%) and North (2.9% versus 7.4%) macro-regions and less than 11 years of schooling (2.2% versus 61.7%) – strata in which we observed a tendency of increasing consumption of ultra-processed foods.

To date, there is record of a single study on changes in eating behavior due to the covid-19 pandemic in Brazil, the ConVid Behavior Research, conducted by the Oswaldo Cruz Foundation (Fiocruz) between April 24 and May 8, 2020. This retrospective study, carried out via the Internet, studied a non-probabilistic sample of 44,062 people residing in Brazil, calibrated to reproduce the sociodemographic distribution of the Brazilian adult population. Its results, for now only published on the Fiocruz ^9^ website, indicate a deterioration in diet quality during the pandemic, evidenced both by the report of a decrease in the usual frequency of consumption of fruits, vegetables and beans and by the report of an increase in the frequency of consumption of ultra-processed foods, such as salty snacks, cookies and chocolates, and frozen meals.

One explanation for the discordant results observed between our study and the ConVid ^9^ research could be the different sociodemographic composition of the populations analyzed, similar to that of the adult Brazilian population, in the Fiocruz research, and underrepresented, in the NutriNet Brasil cohort, of men, older adults, underdeveloped macro-regions and people with less schooling. ConVid results stratified by gender and age are similar to those shown for the whole population, thus indicating that differences in the distribution of these variables may not explain the discrepancies between the two studies ^9^ . ConVid results stratified by macro-region and schooling, which have not yet been disclosed, will be essential to understand the origin of the differences between the two studies and, above all, to confirm the social inequality suggested by our study regarding the consumption of ultra-processed foods during the pandemic.

If confirmed, the trend of increased consumption of ultra-processed foods in the country’s less economically developed regions and by people with less schooling should be cause for concern, as eating these foods increases the risk of obesity, hypertension and diabetes, whose presence increases the severity and lethality of covid-19 ^3^ . Also worrisome is the aggressiveness observed in the advertising of ultra-processed foods during the covid-19 pandemic, either by emphasizing its “contribution to social distancing” and its “anti-monotony effect,” or by promoting the free distribution of products among health professionals ^11^ . The greater impact of this advertising in socially vulnerable strata ^12^ could be one of the factors underlying the trend of increased consumption of ultra-processed foods observed in the Northeast and North regions and among people with less schooling.

On the other hand, the generalized increase in the consumption of vegetables, fruits and legumes during the pandemic observed among participants in the NutriNet Brasil cohort may be an important factor in mitigating the effects of covid-19 ^13^ .

Among the limitations of this study, we highlight the non-probabilistic nature of the sample studied, common in cohort studies, and the still reduced absolute number of participants in some of the country’s macro-regions and in the strata with less schooling, as well as the evaluation of food consumption restricted to healthy and unhealthy food markers. We consider as strengths the study’s focus on dietary changes due to the covid-19 pandemic in Brazil (the only other study to our knowledge is the ConVid research, whose partial results were disclosed only on the Fiocruz website); the total sample size, involving more than 10,000 people; the food questionnaire with objective questions about consumption the day before – less subjected to memory and preferences biases than the questionnaire on usual frequency of consumption employed by the ConVid survey –; and the before and after design, which allowed the same people to answer the questionnaires immediately before and during the covid-19 pandemic in Brazil.
