# Supplementary material for: Dietary changes in the NutriNet Brasil cohort during the covid-19 pandemic
Source: Rev Saude Publica. 2020 Aug 28;54:91. doi: 10.11606/s1518-8787.2020054002950 (PMC7454165; doi:10.11606/s1518-8787.2020054002950)
Supplement: Translation

# Translation: Mudanças alimentares na coorte NutriNet Brasil durante a pandemia de covid-19

**Authors:** Eurídice Martínez Steele, Fernanda Rauber, Caroline dos Santos Costa, Maria Alvim Leite, Kamila Tiemann Gabe, Maria Laura da Costa Louzada, Renata Bertazzi Levy, Carlos Augusto Monteiro

**Affiliations:** I Brasil Universidade de São Paulo. Núcleo de Pesquisas Epidemiológicas em Nutrição e Saúde da USP (NUPENS). São Paulo, SP, Brasil; II Brasil Universidade de São Paulo. Faculdade de Medicina. Departamento de Medicina Preventiva. São Paulo, SP, Brasil; III Brasil Universidade de São Paulo. Faculdade de Saúde Pública. Departamento de Nutrição. São Paulo, SP, Brasil

**Keywords:** Infecções por Coronavirus, Consumo de Alimentos, Alimentos Integrais, Alimentos Industrializados

## Abstract

**OBJETIVO:**

Descrever características da alimentação dos participantes da coorte NutriNet Brasil imediatamente antes e na vigência da pandemia de covid-19.

**MÉTODOS:**

Os dados deste estudo provêm de coorte de adultos criada para investigar prospectivamente a relação entre alimentação e morbimortalidade por doenças crônicas não transmissíveis no Brasil. Para este estudo, foram selecionados os primeiros participantes (n = 10.116) que responderam por duas vezes a questionário simplificado sobre sua alimentação no dia anterior, a primeira vez ao ingressar no estudo, entre 26 de janeiro e 15 de fevereiro de 2020, e a segunda entre 10 e 19 de maio de 2020. O questionário indaga sobre o consumo de marcadores de alimentação saudável (hortaliças, frutas e leguminosas) e não saudável (alimentos ultraprocessados). Comparações de indicadores baseados no consumo desses marcadores antes e na vigência da pandemia são apresentadas para o conjunto da população estudada e segundo sexo, faixa etária, macrorregião de residência e escolaridade. Testes qui-quadrado e testes t foram utilizados para comparar proporções e médias, respectivamente, adotando-se p < 0,05 para identificar diferenças significantes.

**RESULTADOS:**

Para o conjunto dos participantes, identificou-se aumento modesto, porém estatisticamente significante, no consumo de marcadores de alimentação saudável e estabilidade no consumo de marcadores de alimentação não saudável. Esse padrão favorável de mudanças na alimentação com a pandemia se repetiu na maior parte dos estratos sociodemográficos. Padrão menos favorável de mudanças, com tendência de aumento no consumo de marcadores de alimentação saudável e não saudável, foi observado nas macrorregiões Nordeste e Norte e entre pessoas com menor escolaridade, sugerindo desigualdades sociais na resposta à pandemia.

**CONCLUSÕES:**

Caso confirmada, a tendência de aumento no consumo de alimentos ultraprocessados nas regiões economicamente menos desenvolvidas e por pessoas com menor escolaridade preocupa, pois a ingestão desses alimentos eleva o risco de obesidade, hipertensão e diabetes, cuja presença aumenta a gravidade e a letalidade da covid-19.

## INTRODUÇÃO

A pandemia de covid-19, infecção causada pelo novo coronavírus Sars-CoV-2, chegou oficialmente ao Brasil no dia 26 de fevereiro de 2020. Em meados de março, diante do aumento geométrico no número de casos e da inexistência de vacina ou de tratamentos eficazes para a doença, autoridades de saúde dos estados e municípios brasileiros deram início a ações já implementadas em outros países, buscando a diminuição substancial de contatos interpessoais. Essas ações incluíram a paralisação de todas as atividades econômicas não essenciais, o fechamento de escolas e recomendações expressas para que as pessoas ficassem em suas casas o maior tempo possível. Estimativas do dia 10 de maio, feitas com base em dados das operadoras de telefones celulares no Brasil, indicavam que cerca de metade das pessoas seguia as recomendações para ficar em casa^1^ .

É razoável esperar que muitos comportamentos sejam modificados com a pandemia de covid-19, sendo um deles a alimentação. Vários fatores associados à pandemia poderiam influenciar a qualidade da alimentação, tanto positiva quanto negativamente. A maior permanência das pessoas em casa, aliada ao fechamento de bares, restaurantes e outros negócios que servem alimentos ou refeições para consumo no local, faz prever um grande aumento na proporção de refeições feitas na própria residência dessas pessoas, o que seria positivo se considerarmos que a alimentação consumida em casa tende a ser mais saudável do que a consumida fora^2^ . Outro fator que poderia contribuir para a melhoria da alimentação seria uma eventual maior preocupação em consumir alimentos mais saudáveis como forma de aumentar as defesas imunológicas contra uma doença para a qual ainda não há vacina ou tratamento eficaz. Fatores que poderiam levar a comportamentos alimentares menos saudáveis incluiriam a maior dificuldade em obter alimentos frescos, cuja aquisição demanda saídas mais frequentes de casa, e a eventual redução na renda familiar por perda de emprego ou impossibilidade do exercício de determinadas ocupações, limitando a compra de alimentos de maior preço, como são frutas e hortaliças. Destaque-se que a influência de todos esses fatores sobre o comportamento alimentar pode ser modulada por características culturais, educacionais e econômicas das populações e dos indivíduos.

Muitas razões justificam a necessidade de se conhecer o impacto da pandemia de covid-19 sobre o comportamento alimentar da população, incluindo a imprevisibilidade da duração da pandemia e das medidas implantadas para seu controle e a influência que a alimentação tem sobre doenças crônicas não transmissíveis que aumentam a gravidade e letalidade da covid-19^3^ .

Neste artigo descrevemos características da alimentação de participantes da coorte NutriNet Brasil imediatamente antes de a pandemia chegar ao país e durante sua vigência.

## MÉTODOS

Todos os dados deste estudo provêm da coorte NutriNet Brasil, que foi criada para investigar prospectivamente a relação entre padrões de alimentação e morbimortalidade por doenças crônicas não transmissíveis no Brasil^4^ . A participação na coorte é voluntária, e o recrutamento de participantes – pessoas residentes em qualquer estado da Federação com pelo menos 18 anos de idade – foi iniciado em 26 de janeiro de 2020 por meio de convites divulgados por veículos de comunicação de massa de alcance regional e nacional e por redes sociais de influenciadores com dezenas ou centenas de milhares de seguidores em todo o Brasil. A coorte já conta com mais de 75 mil respondentes distribuídos em todos os estados da Federação. Os participantes fazem seu cadastro na plataforma digital do estudo^4^ e, a cada três meses, no mesmo site, respondem a questionários que abordam seu estado de saúde e sua alimentação, além de outras condições que podem influenciar a saúde.

Para este estudo, foram selecionados os primeiros participantes da coorte NutriNet Brasil que responderam por duas vezes a um questionário simplificado sobre sua alimentação no dia anterior, a primeira vez ao ingressar no estudo, entre 26 de janeiro e 15 de fevereiro de 2020 (portanto, antes de a pandemia de covid-19 chegar ao Brasil), e a segunda entre 10 e 19 de maio de 2020 (ou seja, na vigência da pandemia). As informações fornecidas por esses participantes (n = 10.116) foram extraídas do banco de dados da coorte no dia 19 de maio de 2020.

O questionário alimentar simplificado é uma adaptação de instrumento desenvolvido por parte dos autores deste estudo para o sistema de Vigilância de Fatores de Risco e Proteção para Doenças Crônicas por Inquérito Telefônico (Vigitel) do Ministério da Saúde^5^ , que também já foi empregado em duas pesquisas nacionais do Instituto Brasileiro de Geografia e Estatística (IBGE), a Pesquisa Nacional de Saúde (PNS 2019)^6^ e a Pesquisa Nacional de Saúde do Escolar (PeNSE 2019)^7^ . O questionário indaga se, no dia anterior, os participantes consumiram (sim ou não) alimentos selecionados como marcadores de alimentação saudável ou de alimentação não saudável. Apenas a partir do sexto mês de acompanhamento os participantes da coorte NutriNet Brasil responderão recordatórios alimentares de 24 horas completos.

A relação de marcadores de alimentação saudável no questionário simplificado da coorte NutriNet Brasil inclui 29 itens, sendo 18 tipos de hortaliça (alface, acelga, agrião, rúcula, couve, repolho, brócolis, espinafre, qualquer outra verdura, tomate, pepino, cenoura, beterraba, abóbora, abobrinha, berinjela, quiabo e qualquer outro legume), 10 tipos de fruta (banana, laranja ou tangerina, manga, mamão, abacaxi, melancia ou melão, maçã ou pera, uva, açaí, e qualquer outra fruta), além de feijão ou outras leguminosas (lentilha e grão-de-bico).

A relação de marcadores de alimentação não saudável inclui 23 grupos de alimentos ultraprocessados: refrigerante, suco de fruta em caixa ou lata, refresco em pó, bebida achocolatada, bebida à base de chá, iogurte com sabor, salsicha ou hambúrguer ou *nuggets* , presunto ou salame ou mortadela, pão de forma ou de cachorro-quente ou de hambúrguer, margarina, batata frita congelada, maionese ou ketchup ou mostarda, molho pronto para salada, macarrão instantâneo ou sopa de pacote, pizza congelada ou de redes, lasanha congelada ou outro prato pronto comprado congelado, salgadinho de pacote ou batata palha ou biscoito salgado, biscoito doce com ou sem recheio, bolinho de pacote, barra de cereal, sorvete ou picolé de marca, chocolate em barra ou bombom, e cereal matinal açucarado.

Com base nas respostas dadas pelos participantes da coorte sobre o seu consumo alimentar no dia anterior, foram calculados quatro indicadores de alimentação saudável – porcentagem de pessoas que consumiram hortaliça (de qualquer tipo); fruta (de qualquer tipo); feijão ou outra leguminosa; e todos os três itens anteriores – e três indicadores de alimentação não saudável – porcentagem de pessoas que consumiram pelo menos um grupo de alimentos ultraprocessados; cinco ou mais grupos de alimentos ultraprocessados; e número médio de grupos de alimentos ultraprocessados consumidos.

Comparações entre estimativas dos indicadores antes e na vigência da pandemia são apresentadas para o conjunto da população estudada e para estratos definidos por sexo (masculino e feminino), faixa etária (18-39, 40-59 e > 60 anos), macrorregião de residência (Norte, Nordeste, Centro-Oeste, Sudeste e Sul) e escolaridade (< 11, 11 e > 12 anos completos de estudo). Testes estatísticos baseados na distribuição qui-quadrado foram utilizados para comparações de proporções, e testes t para comparação de médias, adotando-se valor de p < 0,05 para identificar diferenças significantes.

## RESULTADOS

A Tabela 1 apresenta características sociodemográficas dos 10.116 participantes da coorte NutriNet Brasil cuja alimentação antes e na vigência da pandemia de covid-19 é descrita neste estudo. Observa-se predomínio do sexo feminino (78,0%), de adultos jovens (51,1%), de residentes na macrorregião Sudeste (61,2%) e de pessoas com 12 ou mais anos de escolaridade (85,1%). O número absoluto de participantes é particularmente reduzido, ficando abaixo de mil, entre idosos (n = 908), residentes nas macrorregiões Centro-Oeste (732) e Norte (290) e pessoas com menos de 11 anos de escolaridade (227).

**Tabela 1:** Distribuição segundo variáveis sociodemográficas. Participantes da Coorte NutriNet Brasil com avaliações do consumo alimentar completas antes a e durante b a pandemia de COVID-19 no Brasil.

Variáveis	N	%
Sexo		
Masculino	2.221	22,0
Feminino	7.895	78,0
Faixa etária (anos)		
18–39	5.174	51,1
40–59	4.034	39,9
> 60	908	9,0
Macrorregião de residência		
Norte	290	2,9
Nordeste	1.122	11,1
Centro-Oeste	732	7,2
Sudeste	6.188	61,2
Sul	1.784	17,6
Escolaridade (anos)		
< 11	227	2,2
11	1.283	12,7
> 12	8.606	85,1
**Total**	**10.116**	**100**

^a^Entre 26 de janeiro e 15 de fevereiro de 2020.

^b^Entre 10 e 19 de maio de 2020.

### Mudanças em Indicadores de Alimentação Saudável

A Tabela 2 apresenta estimativas dos indicadores de alimentação saudável em período imediatamente anterior à chegada da pandemia de covid-19 ao Brasil e durante sua vigência. As estimativas se referem ao conjunto dos participantes estudados e a estratos formados com base em variáveis sociodemográficas.

**Tabela 2:** Indicadores de alimentação saudável antes a e durante b a pandemia de COVID-19 no Brasil segundo variáveis sociodemográficas entre participantes da coorte NutriNet Brasil (n = 10.116).

Variáveis	Consumo no dia anterior de:							
	Hortaliça (%)		Fruta (%)		Feijão ou outra leguminosa (%)		Hortaliça + fruta + leguminosa (%)	
	Antes	Durante	Antes	Durante	Antes	Durante	Antes	Durante
Sexo								
Masculino	85,5	88,7^c^	76,7	81,3^c^	60,6	63,0	43,9	50,5^c^
Feminino	87,8	89,2^c^	78,8	81,9^c^	51,4	53,1^c^	39,1	43,0^c^
Faixa etária (anos)								
18–39	85,2	86,3	72,1	75,7^c^	56,1	56,3	38,8	42,3^c^
40–59	88,9	91,4^c^	83,2	86,9^c^	50,9	54,3^c^	41,1	46,5^c^
> 60	92,5	94,5	92,2	93,5	49,8	54,6^c^	44,4	49,9^c^
Macrorregião de residência								
Norte	84,5	86,9	71,4	76,9	53,4	56,2	36,5	42,8
Nordeste	81,2	85,6^c^	76,6	80,4^c^	63,1	63,6	43,5	47,6
Centro-Oeste	89,7	88,9	81,0	79,8	59,4	58,3	46,7	45,8
Sudeste	87,8	89,5^c^	77,8	82,2^c^	53,6	56,0^c^	40,2	45,9^c^
Sul	89,2	90,2	81,2	82,8	44,6	46,2	36,3	38,1
Escolaridade (anos)								
< 11	81,5	85,5	75,8	81,9	64,3	62,9	42,3	49,8
11	83,4	86,9^c^	73,0	79,9^c^	57,3	61,2^c^	38,4	48,2^c^
> 12	88,1	89,5^c^	79,2	82,0^c^	52,6	54,2^c^	40,4	43,9^c^
Total	87,3	89,1^c^	78,3	81,8^c^	53,5	55,3^c^	40,2	44,6^c^

^a^Entre 26 de janeiro e 15 de fevereiro de 2020.

^b^Entre 10 e 19 de maio de 2020.

^c^p < 0,05

Para o conjunto dos participantes da coorte, os quatro indicadores de alimentação saudável evoluem favoravelmente. Aumentos estatisticamente significantes, ainda que de pequena magnitude, ocorrem para a frequência de consumo no dia anterior de hortaliças (de 87,3% para 89,1%), de frutas (de 78,3% para 81,8%), de feijão ou outras leguminosas (53,5% para 55,3%) e dos três itens anteriores (de 40,2% para 44,6%).

O padrão favorável de mudanças nos quatro indicadores de alimentação saudável se repete na maioria dos estratos, ainda que as variações nem sempre tenham alcançado significância estatística. Mudanças estatisticamente significantes em pelo menos um indicador de alimentação saudável são vistas entre homens e mulheres, em adultos jovens (18–39 anos), de meia idade (40–59 anos) e idosos ( > 60 anos), nas macrorregiões Sudeste e Nordeste e nas categorias de escolaridade intermediária (11 anos) e superior ( > 12 anos).

### Mudanças em Indicadores de Alimentação Não Saudável

Os indicadores de alimentação não saudável antes e na vigência da pandemia de covid-19 no Brasil são apresentados na Tabela 3 .

**Tabela 3:** Indicadores de alimentação não saudável antes a e durante b a pandemia de COVID-19 no Brasil segundo variáveis sociodemográficas entre participantes da coorte NutriNet Brasil (n = 10.116).

Variáveis	Consumo no dia anterior de alimentos ultraprocessados					
	Consumo de pelo menos um grupo (%)		Consumo de cinco ou mais grupos (%)		Número de grupos consumidos (média)	
	Antes	Durante	Antes	Durante	Antes	Durante
Sexo						
Masculino	82,3	83,9	14,3	14,0	2,4	2,4
Feminino	79,3	79,3	10,0	9,4	2,1	2,1
Faixa etária (anos)						
18–39	82,8	83,6	12,9	12,6	2,3	2,3
40–59	78,0	77,8	9,3	8,7	2,0	2,0
> 60	72,8	73,2	6,7	5,6	1,7	1,8
Macrorregião de residência						
Norte	77,6	81,7	14,1	14,1	2,2	2,4
Nordeste	77,9	79,6	8,8	10,9	2,0	2,2^c^
Centro-Oeste	77,5	76,2	7,8	7,4	1,9	1,9
Sudeste	80,4	80,7	11,5	10,5	2,2	2,2
Sul	81,3	80,9	11,3	10,2	2,2	2,2
Escolaridade (anos)						
< 11	86,3	87,2	14,5	15,4	2,5	2,7
11	82,4	83,9	14,1	13,5	2,4	2,4
> 12	79,5	79,6	10,4	9,8	2,1	2,1
Total	80,0	80,3	11,0	10,4	2,1	2,1

^a^Entre 26 de janeiro e 15 de fevereiro de 2020.

^b^Entre 10 e 19 de maio de 2020.

^c^p < 0,05

Para o conjunto dos participantes da coorte, os indicadores de alimentação não saudável praticamente não se modificaram com a pandemia. Assim, a proporção de participantes que consumiram no dia anterior pelo menos um grupo de alimentos ultraprocessados oscilou de 80,0% para 80,3%, e cinco ou mais grupos de 11,0% para 10,4%, enquanto o número médio de grupos consumidos (2,1) permanece inalterado.

O padrão de estabilidade nos indicadores de alimentação não saudável se repete entre homens e mulheres, em todas as faixas etárias, nas macrorregiões Sudeste, Sul e Centro-Oeste e nas categorias intermediária e superior de escolaridade. Entre os participantes da macrorregião Nordeste, observa-se aumento na frequência de consumo de pelo menos um grupo de alimentos ultraprocessados (de 77,9% para 79,6%) e de cinco ou mais grupos (de 8,8% para 10,9%), bem como no número médio de grupos consumidos (de 2,0 para 2,2), sendo a variação neste último caso estatisticamente significante. Ainda que sem alcançar significância estatística, a mesma tendência de aumento no consumo de alimentos ultraprocessados é vista na macrorregião Norte e na categoria inferior de escolaridade, estratos nos quais, por exemplo, o número médio de grupos de alimentos ultraprocessados consumidos aumentou de 2,2 para 2,4 e de 2,5 para 2,7, respectivamente.

## DISCUSSÃO

Com base em questionários sobre o consumo alimentar no dia anterior respondidos por pouco mais de 10 mil participantes da coorte NutriNet Brasil, imediatamente antes e durante a pandemia de covid-19 no país, identificamos aumento modesto, porém estatisticamente significante, no consumo de marcadores de alimentação saudável (hortaliças, frutas e leguminosas) e estabilidade no consumo de marcadores de alimentação não saudável (alimentos ultraprocessados). Esse padrão favorável de mudanças na alimentação com a pandemia se repetiu na maior parte dos estratos da coorte, definidos segundo sexo, idade, macrorregião de residência e escolaridade. Na economicamente menos desenvolvida macrorregião Nordeste, observou-se um padrão menos favorável de evolução, havendo aumentos significativos tanto na frequência de consumo de hortaliças e frutas quanto na quantidade de grupos de alimentos ultraprocessados ingeridos. Ainda que não tenha alcançado significância estatística, possivelmente pelo reduzido número de participantes, esse mesmo padrão de evolução menos favorável da alimentação foi também observado na macrorregião Norte, igualmente menos desenvolvida economicamente, e na categoria inferior de escolaridade, sugerindo desigualdades sociais na resposta do comportamento alimentar à pandemia.

Note-se que, embora a divulgação dos convites para participar da coorte NutriNet Brasil tenha sido feita por veículos de comunicação de massa e redes sociais de alcance regional e nacional, seus primeiros participantes apresentam um perfil sociodemográfico bastante distinto daquele esperado da população brasileira adulta. Na comparação com as frequências esperadas de acordo com o Censo Demográfico de 2010^8^ , destaca-se, além de uma menor proporção de homens (22,0%, nesta pesquisa, contra 48,2% no Censo) e de idosos (9,0% contra 15,3%), uma sub-representação acentuada das macrorregiões Nordeste (11,1% contra 26,6%) e Norte (2,9% contra 7,4%) e da escolaridade inferior a 11 anos (2,2% contra 61,7%), estratos nos quais se observou tendência de aumento no consumo de alimentos ultraprocessados.

Até o presente, há registro de um único estudo sobre mudanças no comportamento alimentar devidas à pandemia de covid-19 no Brasil, a ConVid Pesquisa de Comportamentos, realizada pela Fundação Oswaldo Cruz (Fiocruz) entre 24 de abril e 8 de maio de 2020. Esse trabalho, de natureza retrospectiva e realizado através da internet, estudou uma amostra não probabilística de 44.062 pessoas residentes no Brasil, calibrada de modo a reproduzir a distribuição sociodemográfica da população adulta brasileira. Seus resultados, por ora divulgados apenas no portal da Fiocruz^9^ , indicam uma deterioração da qualidade da alimentação durante a pandemia, evidenciada tanto pelo relato de diminuição na frequência habitual de consumo de frutas, hortaliças e feijão quanto pelo relato de aumento na frequência de consumo de alimentos ultraprocessados, como salgadinhos de pacote, biscoitos e chocolates, e pratos congelados.

Uma das explicações para os resultados discordantes observados entre nosso estudo e a pesquisa ConVid^9^ poderia ser a diferente composição sociodemográfica das populações analisadas, semelhante à da população brasileira adulta, no caso da pesquisa da Fiocruz, e com sub-representação, na coorte NutriNet Brasil, de homens, de idosos, das macrorregiões economicamente menos desenvolvidas e de pessoas com menor escolaridade. Resultados da ConVid estratificados por sexo e idade se assemelham aos mostrados para o conjunto da população nesta pesquisa, não indicando, portanto, que diferenças com relação à distribuição dessas variáveis possam explicar os resultados discordantes entre os dois estudos^9^ . Já os resultados da ConVid estratificados por macrorregião e escolaridade, ainda não divulgados, serão essenciais para entender a origem das diferenças entre as duas pesquisas e, sobretudo, para confirmar a desigualdade social sugerida por nosso estudo quanto ao consumo de alimentos ultraprocessados durante a pandemia.

Caso confirmada, a tendência de aumento no consumo de alimentos ultraprocessados nas regiões economicamente menos desenvolvidas do país e por pessoas com menor escolaridade deve ser motivo de preocupação, pois a ingestão desses alimentos eleva o risco de condições como obesidade, hipertensão e diabetes^10^ , cuja presença aumenta a gravidade e a letalidade da covid-19^3^ . Preocupa também a agressividade observada na publicidade de alimentos ultraprocessados durante a pandemia de covid-19, seja ressaltando sua “contribuição para o distanciamento social” e seu “efeito antimonotonia”, seja promovendo a distribuição gratuita de produtos entre profissionais de saúde^11^ . O maior impacto dessa publicidade em estratos socialmente mais vulneráveis^12^ poderia ser um dos fatores subjacentes à tendência de aumento no consumo de alimentos ultraprocessados observada nas regiões Nordeste e Norte e entre pessoas com menor escolaridade.

Por outro lado, o aumento generalizado no consumo de hortaliças, frutas e leguminosas na vigência da pandemia observado entre os participantes da coorte NutriNet Brasil pode ser um fator importante de mitigação dos efeitos da covid-19^13^ .

Dentre as limitações deste estudo, destacamos a natureza não probabilística da amostra estudada, comum em estudos de coorte, e o ainda reduzido número absoluto de participantes em algumas macrorregiões do país e nos estratos de menor escolaridade, bem como a avaliação do consumo alimentar restrita a marcadores de alimentação saudável e não saudável. Consideramos pontos fortes do estudo o fato de ser um dos primeiros a avaliar as mudanças na alimentação devidas à pandemia de covid-19 no Brasil (o outro único estudo de nosso conhecimento é a pesquisa ConVid, cujos resultados parciais foram divulgados somente no portal da Fiocruz), o tamanho total da amostra, envolvendo mais de 10 mil pessoas, o questionário alimentar com perguntas objetivas sobre o consumo no dia anterior – menos sujeito a vieses de memória e de preferências do que o questionário sobre frequência habitual de consumo empregado pela pesquisa ConVid – e o delineamento antes e depois, que permitiu que as mesmas pessoas respondessem aos questionários imediatamente antes e durante a pandemia de covid-19 no Brasil.
